# Effective Crack Control of Concrete by Self-Healing of Cementitious Composites Using Synthetic Fiber

**DOI:** 10.3390/ma9040248

**Published:** 2016-03-30

**Authors:** Heesup Choi, Masumi Inoue, Sukmin Kwon, Hyeonggil Choi, Myungkwan Lim

**Affiliations:** 1 Department of Civil and Environmental Engineering, Kitami Institute of Technology, Hokkaido 090-8507, Japan; hs-choi@mail.kitami-it.ac.jp (H.C.); m-inoue@mail.kitami-it.ac.jp (M.I.); 2 Public Housing Division, Korea Land & Housing Institute, Daejeon 34047, Korea; sukminkwon@lh.or.kr; 3 Faculty of Environmental Technology, Muroran Institute of Technology, Hokkaido 090-8585, Japan; 4 Department of Architectural Engineering, Songwon University, Gwangju 61756, Korea; limmk79@songwon.ac.kr

**Keywords:** micro-crack, synthetic fiber, PVA, cementitious composite materials, CO_2_micro-bubble, self-healing, Ca(OH)_2_, CO_3_^2−^, CaCO_3_

## Abstract

Although concrete is one of the most widely used construction materials, it is characterized by substantially low tensile strength in comparison to its compression strength, and the occurrence of cracks is unavoidable. In addition, cracks progress due to environmental conditions including damage by freezing, neutralization, and salt, *etc.* Moreover, detrimental damage can occur in concrete structures due to the permeation of deteriorating elements such as Cl^−^ and CO_2_. Meanwhile, under an environment in which moisture is being supplied and if the width of the crack is small, a phenomenon of self-healing, in which a portion of the crack is filled in due to the rehydration of the cement particles and precipitation of CaCO_3_, is been confirmed. In this study, cracks in cementitious composite materials are effectively dispersed using synthetic fibers, and for cracks with a width of more than 0.1 mm, a review of the optimal self-healing conditions is conducted along with the review of a diverse range of self-healing performance factors. As a result, it was confirmed that the effective restoration of watertightness through the production of the majority of self-healing products was achieved by CaCO_3_ and the use of synthetic fibers with polarity, along with the effect of inducing a multiple number of hairline cracks. In addition, it was confirmed that the self-healing conditions of saturated Ca(OH)_2_ solution, which supplied CO_2_ micro-bubbles, displayed the most effective self-healing performance in the surface and internal sections of the cracks.

## 1. Introduction

Concrete and cementitious construction materials are essential construction materials for buildings, civil engineering, and general construction in modern society, and it is deemed that the development of construction materials that can completely substitute for concrete is very problematic, even in the future. Meanwhile, since concrete is a material with tensile strength that is substantially low in comparison to its compression strength, the occurrence of cracks in concrete structures is unavoidable. In the case of Japan, cracks generated by the aforementioned reasons and having a width less than the allowable level are determined to impart no major effects on, or problems in, the durability of a structure [1]. However, although such micro-cracks in concrete are not in themselves a threat to the safety performance of structures, deteriorating elements such as CO_2_ and Cl^−^ are permeated into the body of the concrete by the micro-cracks, and in turn increase the water permeability, which is an index of durability evaluation [2]. In addition, repetitive permeation of such deteriorating elements expands the width of the cracks, thereby accelerating the deterioration of the concrete [3]. Due to this process, it is determined that there is an increased likelihood of a detrimental effect on the safety performance of concrete structures [4,5]. Accordingly, there is a need for the prevention of micro-cracks in concrete structures at a more fundamental stage.

Meanwhile, under a moist environment, the phenomenon of filling in of portions of the cracks in concrete due to rehydration of the cement particles and precipitation of CaCO_3_ or calcite, particularly when the width of the crack is small, is being confirmed [6]. The self-healing products are closely related to hydrates such as C-S-H hydrate, ettringite, and calcium hydroxide, *etc.* [7,8], along with the calcium carbonate newly generated from the surfaces of the crack [3]. The self-healing mechanism of concrete generates CaCO_3_, which is a carbon compound that does not dissolve well in water, through the reaction between Ca^2+^ in the concrete with the CO_3_^2−^ dissolved in the water. This phenomenon leads to the reclamation or in-filling of the cracked portions [9]. The following are the crystalline reaction Equations (1)–(3) of calcite:

H_2_O + CO_2_ ⇔ H_2_CO_3_ ⇔ H^+^ + HCO_3_^−^ ⇔ 2H^+^ + CO_3_^2−^ (1)

Ca^2+^ + CO_3_^2−^ ⇔ CaCO_3_ {pH_water_ > 8} (2)

Ca^2+^ + HCO_3_^−^ ⇔CaCO_3_ + H^+^ {7.5 < pH_water_ < 8} (3)

Also, various self-healing approaches attempt to promote autogenous healing, such as the use of bacteria, crystalline admixtures, and superabsorbent polymers [10,11,12,13,14]. This self-healing phenomenon can delay the permeation of Cl^−^ by reducing the cracks in the concrete, and lead to a reduction in the permeability coefficient by partially restoring the permeability, which had been markedly increased due to the cracks [2,15]. In addition, it can almost fully restore the elastodynamic coefficient of concrete in which deterioration had occurred due to freeze–thawing, and also partially restore the deteriorated strength of the concrete [16]. Therefore, if a crack generated by the aforementioned causes can be restored through self-healing, it is possible to manage the progress of cracks effectively at the initial stage of their occurrence and, ultimately, to achieve the suppression of degradation in the safety performance of concrete structures. In addition, self-healing can make substantial contributions toward the ease of maintenance of concrete structures and ensure a reduction in environmental load, along with the prolongation of the lifespan of structures.

Existing research reports that engineered cementitious composites (ECC) with multiple fine cracking have the greatest potential for the practical achievement of self-healing in concrete [17,18]. Especially, ordinary concrete restores cracks of 50 µm [19] and 30 µm [14] width through self-healing [9]. In addition, the self-healing performance can be made more efficient by effectively dispersing the cracks and thereby substantially reducing the width of the cracks generated. This can be achieved by mixing synthetic fibers such as polyvinyl alcohol (PVA), polyethylene (PE) and polypropylene (PP), *etc.* into the concrete [20,21]. In particular, it has been confirmed that by using PVA fibers with polarity-induced OH^−^ radicals, much better self-healing, along with effective dispersion of cracks, can be achieved even for cracks with a width of more than 0.1 mm where the permeation of CO_2_ gas and chloride ion (Cl^−^) is a concern [22].

This study assessed the composite self-healing performance of the cementitious materials to which a diverse range of synthetic fibers had been added, and the effect on micro-cracks with a width of more than 0.1 mm, which can result in serious degradation of the durability of the concrete structure. After manufacturing the specimen using synthetic fiber-reinforced cementitious composite materials, micro-cracks were induced in the specimen by applying tensile force. This was followed by the measurement of the changes in the physical characteristics of the specimen, structural changes in the surface and internal sections of the cracked sections, and the types and quantities of products arising from the self-healing of the introduced micro-cracks. In addition, it was concurrently attempted to identify the optimal self-healing conditions by inducing self-healing under an extensive range of conditions.

## 2. Materials and Method

### 2.1. Materials

**Figure 1:**
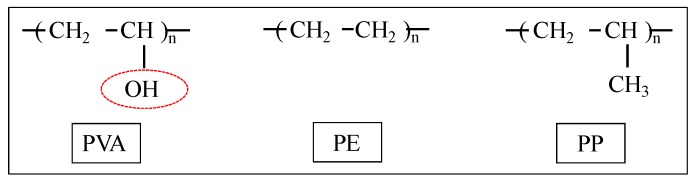
Characteristic part of the chemical components of each fiber.

The mixture proportions of the mortar are summarized in Table 1. Portland cement (C, density: 3.16 g/cm^3^, mean diameter: 10 μm), quartz sand as the fine aggregate (S, surface-dry density: 2.61 g/cm^3^, mean diameter: 180 μm), and a high-performance water reducing agent as an admixture (SP, density: 1.05 g/cm^3^, main constituent: polycarboxylate-based superplasticizer) were used. As for the synthetic fibers, PVA (fiber diameter: 40 μm, fiber length: 12 mm, density: 1.3 g/cm^3^) and polyethylene (PE) (fiber diameter: 12 μm, fiber length: 12 mm, density: 0.97 g/cm^3^) and polypropylene (PP) (fiber diameter: 65 μm, fiber length: 12 mm, density: 0.91 g/cm^3^) were used. The properties of the employed fibers are presented in Table 2. The chemical components of the employed synthetic fibers are characterized by polar groups as shown in Figure 1. PVA has the highest polarity strength owing to the OH radical (indicated by the circle), whereas PE and PP have no polarity strength.

### 2.2. Specimen Overview

**Figure 2:**
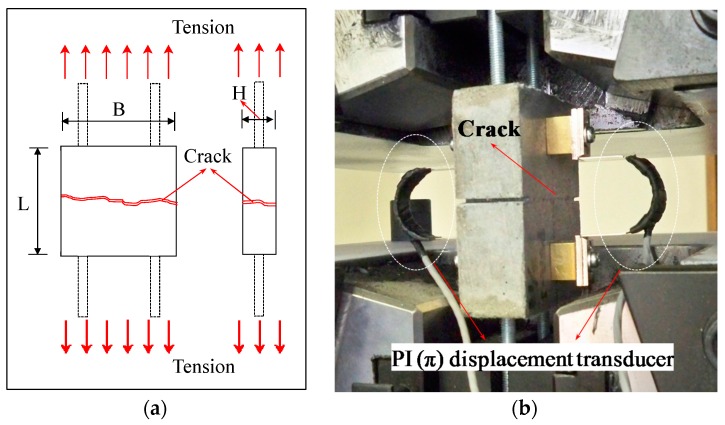
Specimen overview. (**a**) Geometry of the specimen; (**b**) Direction of the load during the tensile test. PI: π.

The dimensions of the specimens were 85 × 80 × 30 mm (L × B × H). Two specimens were fabricated for each series. After mixing the mortar with the fibers, water curing was performed in a tank at 20 °C for 28 days. A universal testing machine (UTM) was used to apply a tensile load at a speed of 0.2 mm/min, and the crack width was adjusted so that the displacement of the PI (π) displacement transducer would be about 0.3 mm. Figure 2a,b show the mimetic diagram of the specimens, used in crack introduction, and the tensile load test.

### 2.3. Experimental Method

**Figure 3:**
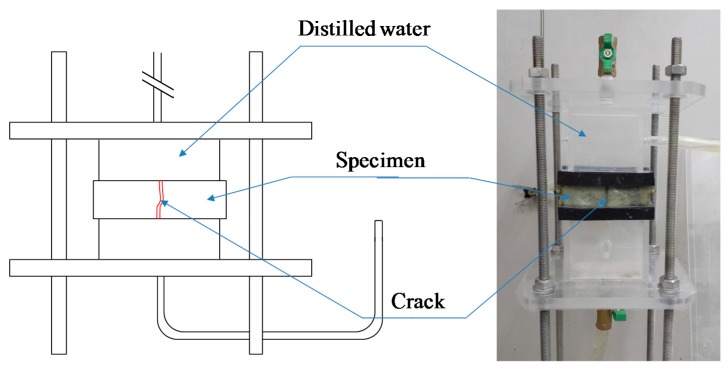
Apparatus used in the water permeability tests [23].

The order and evaluation items of the experiment for the assessment of the composite self-healing performance of the concrete, in accordance with the addition of synthetic fibers, are as follows. First, in Step A (prior to self-healing), an analysis of the permeability coefficient immediately following the introduction of the cracks by the tensile loading test, observation of the internal sections of cracks using microfocus X-ray computed tomography (CT) scans, and an analysis of the types and quantities of the hydrates prior to self-healing using the thermo gravimetric-differential thermal analysis (TG-DTA) measurement, were executed. In Step B (After self-healing), a comparison was made using the method applied in Step A in order to evaluate the changes in the permeability of each of the specimens due to self-healing and changes in the structure within the cracks, a quantitative evaluation of the self-healing precipitated substances was undertaken. Especially, the coefficient of water permeability was calculated by the water flow speed through the plate specimen in Step A. Then, all specimens were kept for 7 days in a water tank at 20 °C. After the curing for self-healing, the coefficient of water permeability was evaluated by the water flow speed through the plate specimen in Step B. The apparatus used in the water permeability tests in this study is shown in Figure 3 [23]. Furthermore, in Step B, the self-healing characteristics and the types of precipitated substances at the surface section of the cracks were assessed by optical microscopic observation and Raman spectroscopy analysis. The experimental factors and conditions are summarized in Table 3. As the conditions for self-healing in this experiment, two conditions were used in existing research; namely, water (tap water) that supplied CO_2_ micro-bubbles (W + MB) and saturated Ca(OH)_2_ solution (Ca + MB) were employed. The water temperature was adjusted to 20°C for both, and the pH to 6.0 and 8.5, respectively, for the evaluation of the self-healing performance of each of the specimens in accordance with each of the aforementioned conditions of self-healing, with the setting of the period of self-healing to 7 days. It is thought that the Ca^2+^ in a saturated Ca(OH)_2_ solution promotes self-healing and that there is a possibility of promoting the generation of self-healing precipitated substances through an increase in the quantity of CO_3_^2−^ supplied by CO_2_ micro-bubbles at the time of self-healing [24,25]. Therefore, this was applied in this study to maximize the self-healing performance.

## 3. Results and Discussion

### 3.1. Permeability Coefficient

**Figure 4:**
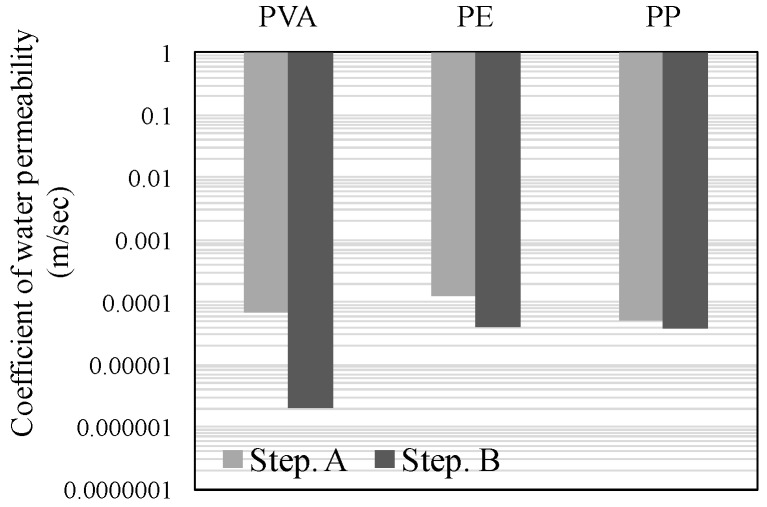
Permeability coefficientof Water + Micro bubble (W + MB).

**Figure 5:**
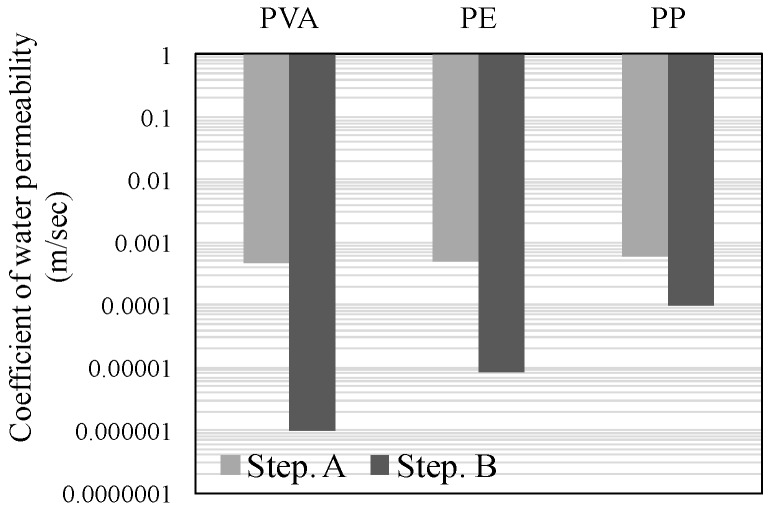
Permeability coefficientof Ca(OH)_2_ + Micro-bubble (Ca + MB).

**Figure 6:**
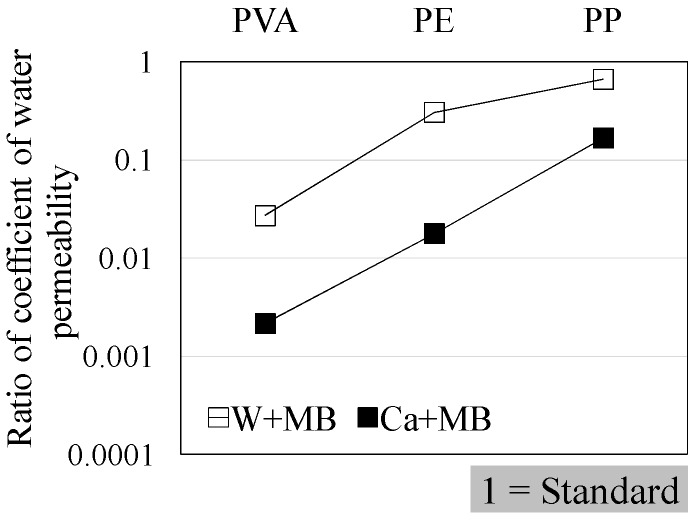
Comparison of permeability coefficient ratio.

Figure 4, Figure 5 and Figure 6 illustrate the results of the water permeability test. Here, Figure 4 and Figure 5 display the permeability coefficients of each of the fiber series prior to, and following self-healing in accordance with the conditions of self-healing. Figure 6 displays the permeability coefficient ratio for each of the fiber series computed on the basis of the permeability coefficient values of Step A. In addition, the lower permeability coefficient of each of the graphs signifies improvement in the resistance to permeability.

The results of the experiment, (W + MB) in Step B, when compared with that in Step A, displayed the trend of an increase in the resistance to permeability by about 40-fold for PVA, 3.5-fold for PE, and 1.5-fold for PP (Figure 4). Meanwhile, (Ca + MB) in Step B, when compared with that in Step A, displayed the trend of an increase in the resistance to permeability by about 460-fold for PVA, 60-fold for PE, and 6-fold for PP (Figure 5). In addition, in the comparison of the permeability coefficient following self-healing as illustrated in Figure 6, (Ca + MB), in comparison to (W + MB), displayed the trend of improvement in the resistance to permeability by approximately 15-fold for PVA, 17-fold for PE, and 4-fold for PP. From the aforementioned results, it can be discerned that the resistance to permeability is improved in the order of PVA > PE > PP, regardless of the conditions of self-healing. In particular, PVA with OH^−^ radical displayed a more effective self-healing performance, and it was confirmed that the conditions of (Ca + MB) were more advantageous than the conditions of (W + MB) for the promotion of self-healing performance. Therefore, for micro-cracks with a width of more than 0.1 mm, for which substantial permeation of deteriorating elements from the external into the internal sections of the concrete was anticipated, it was deemed that the generation and precipitation of the self-healing substances were promoted due to the mixing of the PVA fiber with the OH^−^ radical, along with the enhancement of the conditions of self-healing by the saturated Ca(OH)_2_ solution (Ca^2+^) that contained CO_2_ micro-bubbles (CO_3_^2−^) [8,24].

### 3.2. Microscopic Review of the Crack Section Due to Self-Healing

#### 3.2.1. Surface Section of the Cracks

Microscopic observation of the surface section of the cracks was executed using an optical microscope and Raman spectroscopy in order to check the precipitated substances generated by the self-healing at the surface of the cracks. Since a white-colored precipitated substance was observed at the surface section of the cracks, which exhibited self-healing for both the (W + MB) and (Ca + MB), microscopic observation of the surface section of the cracks was made for the presence of self-healing (Ca + MB), which was determined to be advantageous in the promotion of self-healing on the basis of the outcomes described in Section 3.1.

**Figure 7:**
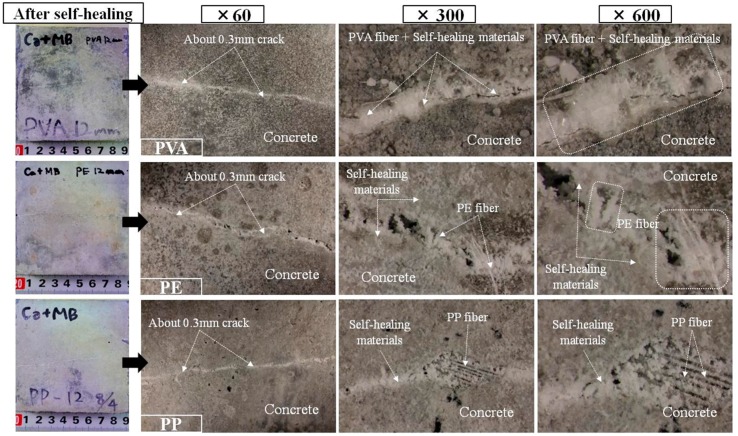
Surface section of the cracks of each of the specimens(Ca + MB).

The results of the optical microscopic observation are illustrated in Figure 7. In the case of PVA, the surface of cracks was completely blocked by a white-colored precipitated substance, obscuring the observation of the configuration of the fibers themselves. In the case of PE, a white-colored precipitated substance was observed in portions of the surface of the cracks, with a substantial quantity attached to the area around the fibers. Meanwhile, in the case of PP, in comparison to PVA and PE, there was almost no attachment of a white-colored precipitated substance to the area around the fibers, and the fibers themselves could be clearly distinguished under observation. From these results it can be proposed that the white colored substance is precipitated by self-healing and that the promotion of self-healing would be possible in the order of PVA > PE > PP in all of the test specimens.

**Figure 8:**
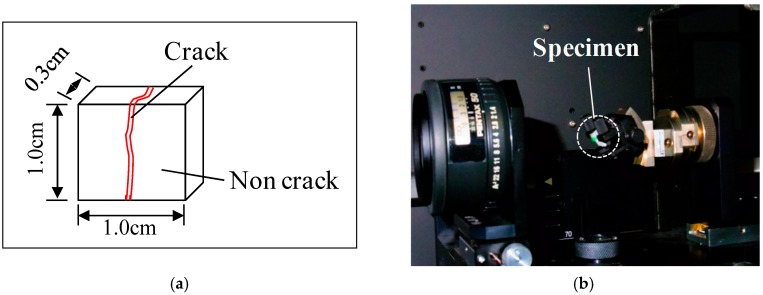
Experimental overview of the Raman spectroscopy analysis. (**a**) Geometry of the specimen; (**b**) Raman spectroscopy.

**Figure 9:**
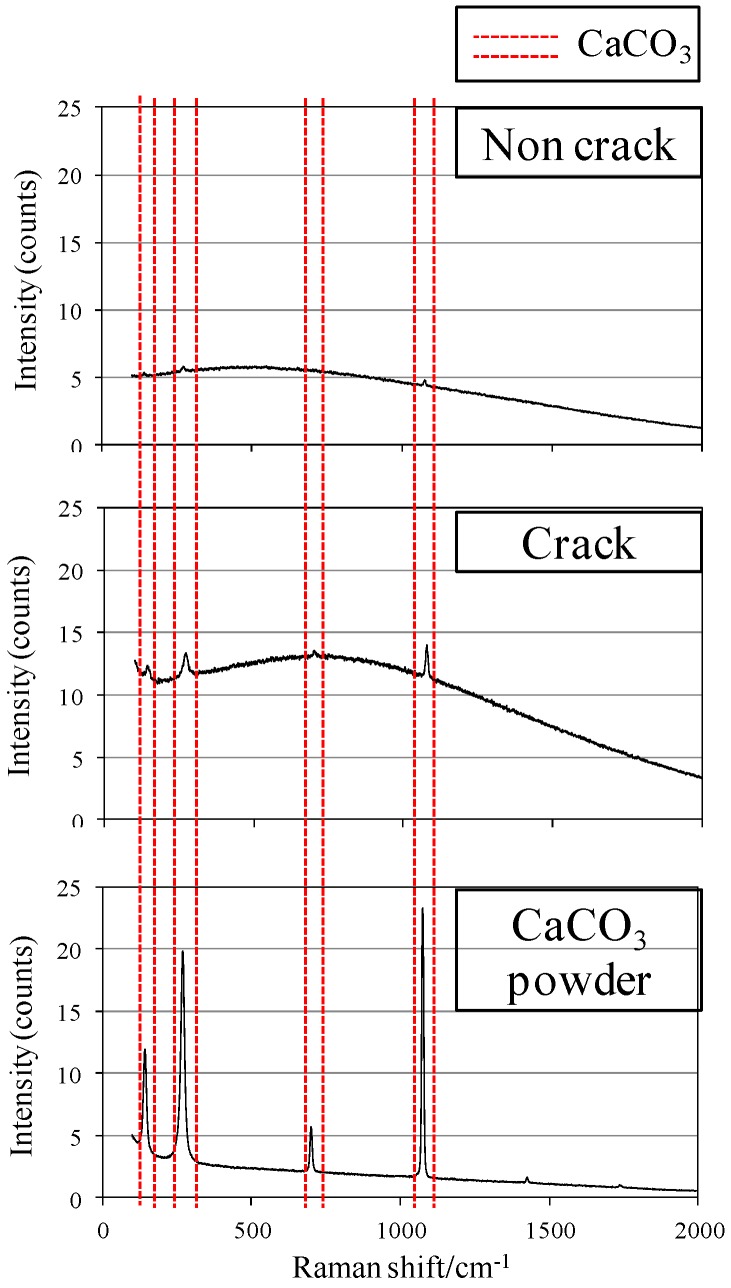
PVA (Ca + MB).

Figure 8a,b show the experimental overview of the Raman spectroscopy analysis, and Figure 9 displays the experimental results of Raman spectroscopy. A comparison was made of the locations of the occurrence of the peak of the wave generated by the laser at the crack section of PVA specimen to which the white-colored precipitated substance was attached, and that at the sections without cracks. With the peak of the wave indicating CaCO_3_ powder as the subject of comparison, it can be seen that there was almost no peak in the wave that coincided with that of the CaCO_3_ in the sections without cracks. However, the peak of the wave in the crack section accurately coincides with the peak of the wave of CaCO_3_ powder. Accordingly, it is concluded that the majority of the white-colored precipitated substance was CaCO_3_ generated during self-healing.

#### 3.2.2. Internal Aspect of the Cracks

**Figure 10:**
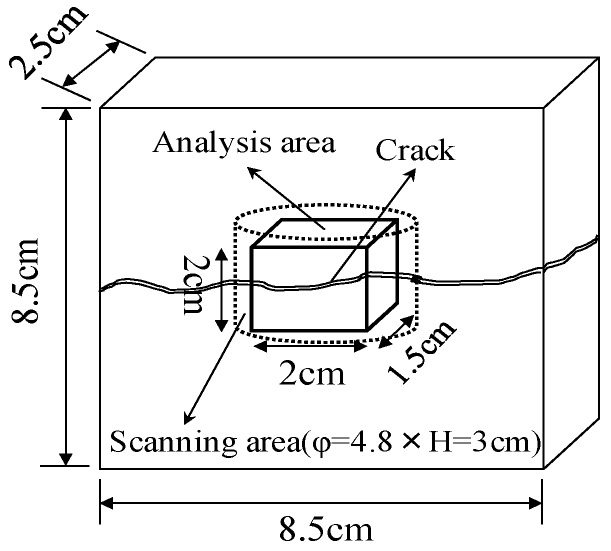
Area of X-ray computed tomography (CT) scanning.

**Figure 11:**
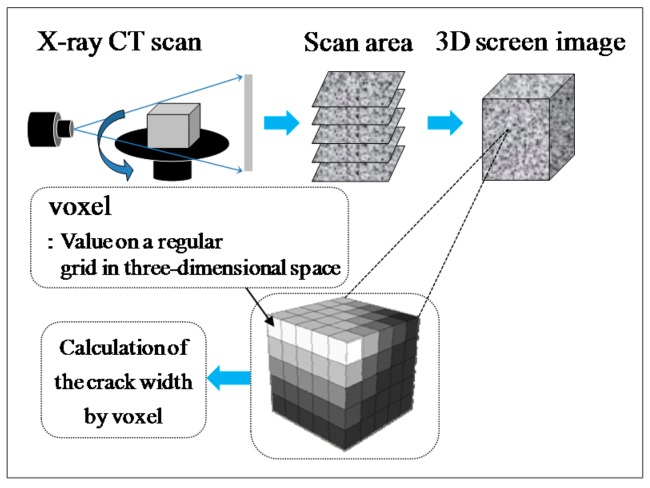
Method of calculating the crack width [26].

**Figure 12:**
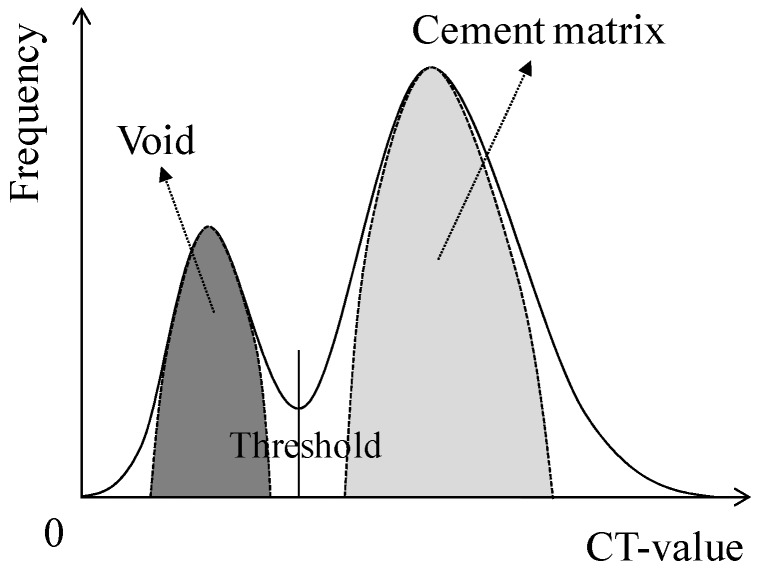
Conceptual diagram of the frequency of CT-value.

**Figure 13:**
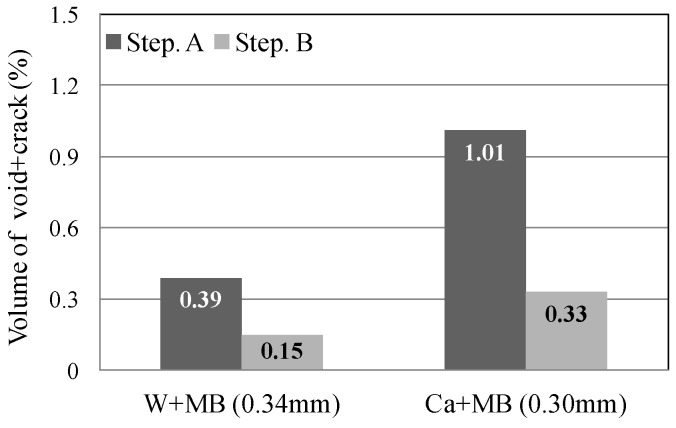
PVA fiber.

**Figure 14:**
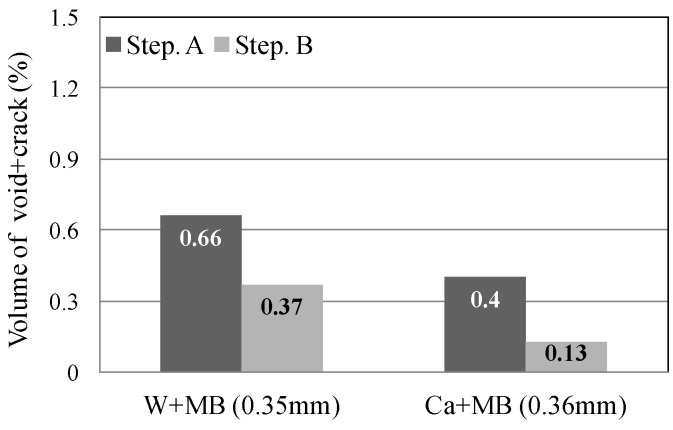
PE fiber.

**Figure 15:**
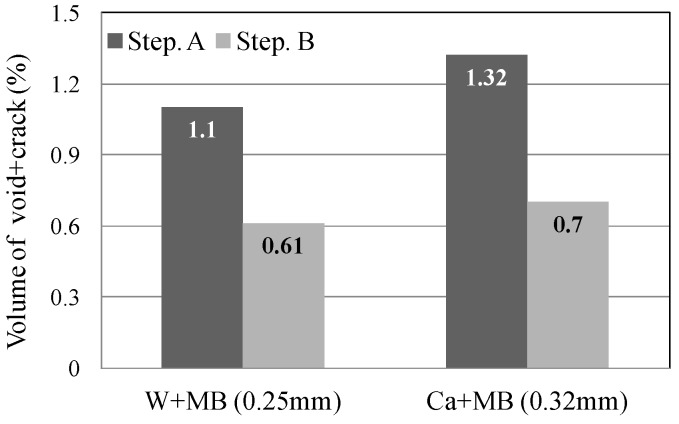
PP fiber.

**Figure 16:**
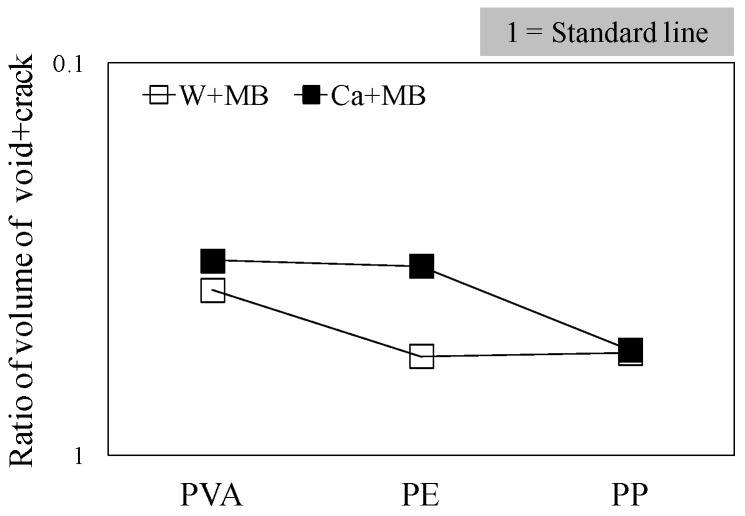
Ratio of volume of (void + crack).

In this experiment, the internal sections of the cracks in the specimens were observed using microfocus X-ray CT scans in order to assess the status of the progress of self-healing within the cracks. As the conditions of the experiment, an X-ray of 200 kV and 100 μA was used. As illustrated in Figure 10, a screen image interpretation domain of the X-ray CT scan was set. Here, the 3D screen image of each of the specimens obtained through the X-ray CT scan was composed of a voxel, and the width of the cracks and the volume of the crack sections of each of the specimens were computed using this voxel (Figure 11) [26]. In addition, Figure 12 illustrates the conceptual diagram of the histogram of the luminance (CT-value) and frequency of the 3D screen image. Here, the boundaries of the luminance (CT-value) of the void and substance (cement matrix) were clearly distinguished through the regular distribution of each of the peaks. The crack section prior to the self-healing had the same density as the void, and the volume of the void was computed. Moreover, regarding the crack section, the changes in the volume of the void section prior to, and following, the self-healing were compared and assessed using the difference in the densities of the void section and the section filled in by the precipitated substance. Figure 13, Figure 14 and Figure 15 illustrate the changes in the volume of the void sections prior to, and following, self-healing in accordance with the width and the conditions of self-healing of each of the specimens. In addition, the graph in Figure 16 compares the ratio of changes in the volume of the void section following self-healing with the volume of the void section of Step A as the reference. In the PVA series in Figure 13, the volume of the void section was reduced by approximately 62% for the (W + MB) and by 67% for the (Ca + MB) in Step B in comparison to those in Step A. In the case of the PE series in Figure 14, the volume of the void section was reduced by approximately 44% for the (W + MB) and 67% for the (Ca + MB) in Step B in comparison to those in Step A. In the case of the PP series seen in Figure 15, there was the trend of reduction in the volume of the void section by approximately 44% for the (W + MB) and 46% for the (Ca + MB) in Step B in comparison to those in Step A. Meanwhile, in terms of the ratio of the changes in the volume of the void section of all the specimens as illustrated in Figure 16, although there was almost no difference between PE and PP in the case of (W + MB), PVA displayed the tendency of a reduction in the ratio of the volume of the void section by approximately 1.5-fold in comparison to that of PE and PP. In addition, in the case of (Ca + MB), PVA and PE displayed the tendency of reduction in the ratio of volume of the void section by about 1.6-fold in comparison to PP.

Based on the above results, the performance of self-healing by each of the fibers was found to be in the order of PVA ≥ PE > PP. From the existing researches [13,27], internal cracks were blocked by self-healing with a significantly longer time. However, in this study, it was determined that the self-healing performance of internal cracks can be maximized by concurrently and appropriately using the (Ca + MB) conditions of supplying the Ca^2+^ and CO_3_^2−^ necessary in self-healing along with the use of PVA fiber with an OH^−^ radical [28].

### 3.3. Chemical Evaluation of the Precipitated Substances of Self-Healing

**Figure 17:**
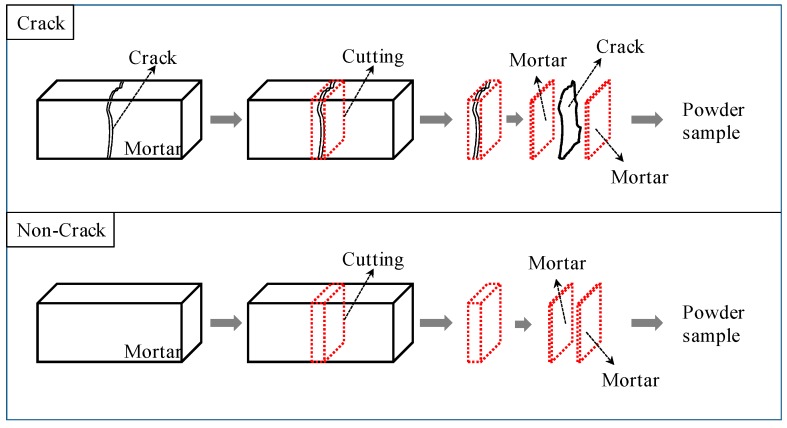
Sampling of specimen for thermo gravimetric-differential thermal analysis (TG-DTA).

For the types of, and quantitative evaluation of, the quantities of the substance precipitated by self-healing at the surface and internal sections of the cracks, a comparison and evaluation was made by means of TG-DTA for the quantitative changes in Ca(OH)_2_ and CaCO_3_ prior to and following the self-healing for each of the fiber series. Also, TG-DTA samples in the experiment were collected from each specimen, as shown in Figure 17.

**Figure 18:**
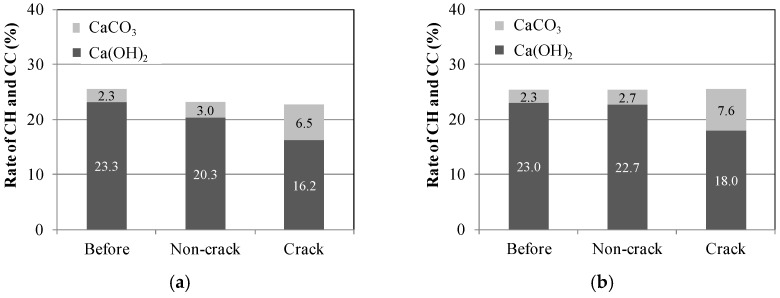

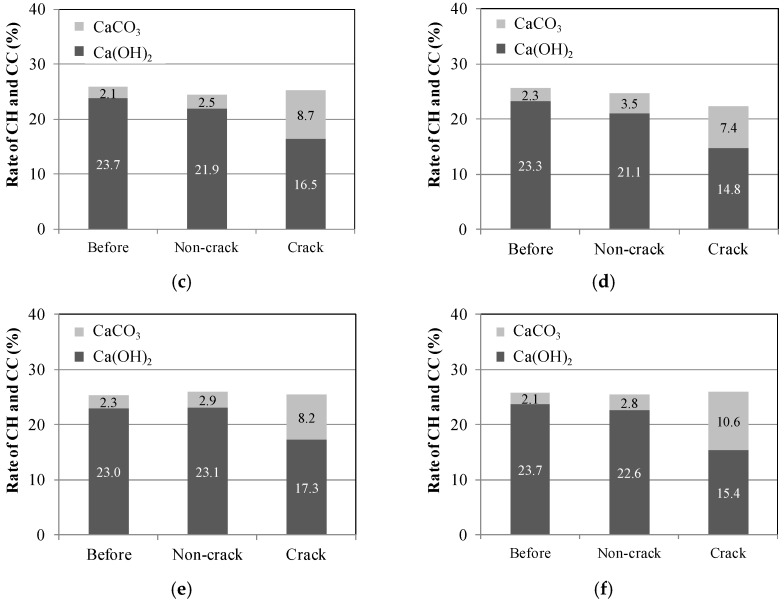
Comparison of the self-healing precipitated substances. (**a**)PP (W + MB); (**b**) PE (W + MB); (**c**) PVA (W + MB); (**d**) PP (Ca + MB); (**e**) PE (Ca + MB); (**f**) PVA (Ca + MB).

Figure 18 illustrates the outcome of the TG-DTA experiment on the sections without cracks (Non-crack) and the sections with cracks (Crack) prior to, and following, the self-healing for each of the fibers and the conditions of self-healing. The quantity of the Ca(OH)_2_ displayed a tendency to decrease, while that of CaCO_3_ increased for the Crack sections in comparison to those prior to self-healing (Before) and to those of the Non-crack sections in all the fiber series. Here, quantities of CaCO_3_, presumed to be the substance precipitated by self-healing, increased in the order of PVA > PE > PP. In particular, in the case of the PVA series, the quantity of Ca(OH)_2_ in the crack section was reduced by about 7%, while the quantity of CaCO_3_ was increased by about 6% in comparison to those prior to self-healing. The tendency of a reduction in the quantity of Ca(OH)_2_ and an increase in the quantity of CaCO_3_ was also displayed in comparison to the Non-crack sections following self-healing for (Figure 18c) (W + MB). In addition, for (Figure 18f) (Ca + MB), there was substantial increase in the quantity of CaCO_3_ when PVA was used in comparison with the use of PP and PE, and compared to (W + MB). From such results, it was determined that self-healing was further promoted. Therefore, as the results of the aforementioned comparison and evaluation of the types and the quantities of the substances precipitated due to self-healing, it was possible to generate a greater quantity of precipitated substances of self-healing under the condition of (Ca + MB) in comparison to that under the condition of (W + MB) for each of the fiber series (PVA, PE, and PP). Moreover, it was determined that the majority of this precipitated substance was CaCO_3_. In addition, it was possible to achieve not only self-healing of the cementitious composite materials, but also improvement of self-healing performance by synthetic fibers by using PVA with the OH^−^ radical. Therefore, it was determined that more effective self-healing performance is possible for micro-cracks with a width of more than 0.1 mm for which substantial permeation of deteriorating elements from the external sections into the internal sections of the concrete is anticipated.

## 4. Conclusions

This study aimed to assess the changes in the physical properties and structure of the surface and internal sections of cracks during composite self-healing by cementitious composite materials and synthetic fibers. This included changes in the type and quantity of the precipitated substance, and the optimal conditions of self-healing. The study examined the effective dispersion of cracks in the cementitious composite materials reinforced with synthetic fiber, and demonstrated self-healing of cracks of approximately 0.3 mm width. The changes in the structure, observations of the surface and internal sections of the cracks using the permeability coefficient, optical microscope, and X-ray CT scan, and experimental results of the comparison and evaluation of the types and quantities of the precipitated substance by using Raman spectroscopic analysis and TG-DTA, are summarized as follows:

(1) It was confirmed that self-healing of cementitious composite materials alone and cementitious composite materials mixed with synthetic fiber with polarity progressed not only on the surface of the cracks but also in the internal sections of the cracks.

(2) Composite self-healing of the cementitious composite materials and synthetic fiber with polarity generates a precipitated substance on the surface and internal sections of the micro-crack, and at this time, it was confirmed that the majority of the precipitated substance is CaCO_3_.

(3) It is possible to restore micro-cracks with width of more than 0.1 mm, for which substantial permeation of deteriorating elements from the external into the internal sections of the concrete is anticipated, by mixing synthetic fiber with the concrete. In particular, PVA fiber with polarity is able to restore water tightness and precipitate large quantities of self-healing substances to a greater extent than the PE and PP fibers. Accordingly, it is determined that PVA is able to achieve more effective self-healing performance.

(4) Regarding the optimal condition of self-healing, it was confirmed that applying conditions of saturated Ca(OH)_2_ solution plus CO_2_ micro-bubbles, which supply the Ca^2+^ and CO_3_^2−^ necessary for self-healing, is effective in promoting self-healing performance. This is due to the generation of the self-healing substance and an increase in the quantity of precipitation available for crack reclamation.

## Figures and Tables

**Table 1:** Mixture proportions of the mortar.

Type	S/C (wt.%)	W/C (wt.%)	SP/C (wt.%)	Fiber (vol.%)
PVA	0.4	0.3	0.4	1.2
PE			0.45	
PP			0.3	

Note: wt.: Weight; vol.: Volume; S: Quartz sand; C: Portland cement; W: Water; SP: High-performance water reducing agent; PVA: Polyvinyl alcohol; PE: Polyethylene; PP: Polypropylene.

**Table 2:** Properties of employed fibers.

Type	Type of Fiber	Density (g/cm^3^)	Tensile Strength (N/mm^2^)	Length (mm)	Diameter (μm)
PVA	Polyvinyl alcohol	1.30	1600	12	40
PE	Polyethylene	0.97	2580	12	12
PP	Polypropylene	0.91	500	12	65

**Table 3:** Experimental factors and conditions.

Experimental Factors			Conditions
Fiber			PVA, PE, PP
Self-healing	Water + Micro-bubble	(W + MB)	pH 6.0
	Ca(OH)_2_ + Micro-bubble	(Ca + MB)	pH 8.5
Temperature			20 °C
Crack (Target of crack width: 0.3 mm)			Tensile load
Period of self-healing			7 Days

Note: W + MB: Water + Micro-bubble; Ca + MB: Ca(OH)_2_ + Micro-bubble.
